# 11β-hydroxysteroid dehydrogenase-1 deficiency alters the gut microbiome response to Western diet

**DOI:** 10.1530/JOE-16-0578

**Published:** 2016-12-16

**Authors:** Jethro S Johnson, Monica N Opiyo, Marian Thomson, Karim Gharbi, Jonathan R Seckl, Andreas Heger, Karen E Chapman

**Affiliations:** 1 Computational Genomics Analysis and TrainingMedical Research Council-Functional Genomics Unit, Department of Physiology, Anatomy and Genetics, University of Oxford, Oxford, UK; 2 University/BHF Centre for Cardiovascular ScienceQueen’s Medical Research Institute, Edinburgh, UK; 3 Edinburgh GenomicsAshworth Laboratories, University of Edinburgh, Edinburgh, UK

**Keywords:** glucocorticoids, 11β-HSD, diet, gut microbiome

## Abstract

The enzyme 11β-hydroxysteroid dehydrogenase (11β-HSD) interconverts active glucocorticoids and their intrinsically inert 11-keto forms. The type 1 isozyme, 11β-HSD1, predominantly reactivates glucocorticoids *in vivo* and can also metabolise bile acids. 11β-HSD1-deficient mice show altered inflammatory responses and are protected against the adverse metabolic effects of a high-fat diet. However, the impact of 11β-HSD1 on the composition of the gut microbiome has not previously been investigated. We used high-throughput 16S rDNA amplicon sequencing to characterise the gut microbiome of 11β-HSD1-deficient and C57Bl/6 control mice, fed either a standard chow diet or a cholesterol- and fat-enriched ‘Western’ diet. 11β-HSD1 deficiency significantly altered the composition of the gut microbiome, and did so in a diet-specific manner. On a Western diet, 11β-HSD1 deficiency increased the relative abundance of the family *Bacteroidaceae*, and on a chow diet, it altered relative abundance of the family *Prevotellaceae*. Our results demonstrate that (i) genetic effects on host–microbiome interactions can depend upon diet and (ii) that alterations in the composition of the gut microbiome may contribute to the aspects of the metabolic and/or inflammatory phenotype observed with 11β-HSD1 deficiency.

## Introduction

The gut is increasingly recognised as an important source of bacterially derived signals that have the potential to influence host physiology, including via the endocrine system (Evans *et al*. 2013, Brown & Hazen 2015). Conversely, dietary or other metabolic perturbations can alter the composition of the gut microbiota in a manner that correlates with disease states (Giongo *et al*. 2011). Although increased understanding of these host–microbiome interactions promises to identify many novel therapeutic targets for the prevention and treatment of disease, at present, the molecular mechanisms that underpin such interactions remain largely uncharacterised.

The enzyme 11β-hydroxysteroid dehydrogenase type 1 (11β-HSD1) contributes to intracellular glucocorticoid action by catalysing the conversion of inactive to active forms of the principal glucocorticoids found in humans (cortisone to cortisol) and mice (11-dehydrocorticosterone to corticosterone). Whilst widely distributed, 11β-HSD1 regulates intracellular glucocorticoid levels in a localised and tissue-specific manner, largely independent of circulating cortisol/corticosterone levels (Chapman *et al*. 2013). It is therefore an important contributor to glucocorticoid modulation of immunological and metabolic pathways. Tissue levels of 11β-HSD1 are elevated in adipose tissue in metabolic disease and in hippocampus in age-related cognitive decline. Inhibition of 11β-HSD1 is a potential therapeutic target for treatment of a range of conditions, including type 2 diabetes-metabolic syndrome, atheromatous coronary artery disease and age-related cognitive decline (Anderson & Walker 2013, Chapman *et al*. 2013).

In mice, inhibition of, or deficiency in, 11β-HSD1 is protective against the adverse metabolic and pro-inflammatory effects of a high-fat diet (Morton *et al*. 2004, Wamil *et al*. 2011) and improves insulin sensitivity/glucose homeostasis in models of type 2 diabetes (Morton *et al*. 2004). 11β-HSD1 deficiency/inhibition also reduces atherosclerosis in atherosclerosis-prone *Apoe*^−/−^ mice fed a cholesterol and fat-enriched ‘Western’ diet, attributed in part to reduced cholesterol accumulation in macrophages (García *et al*. 2013, Kipari *et al*. 2013). Consistent with the widely recognised role of glucocorticoids within the immune system, 11β-HSD1 deficiency or inhibition in mice increases the severity of acute inflammatory responses (Coutinho *et al*. 2012) and modulates macrophage phenotype (Gilmour *et al*. 2006, Zhang & Daynes 2007, McSweeney *et al*. 2010), the latter, potentially an important determinant of gut microbial composition (Wu & Wu 2012). However, the relationship between 11β-HSD1 activity and composition of the gut microbiota has not been examined.

In addition, there is increasing evidence that 11β-HSD1 both influences and is, in turn, directly influenced by bile acid homeostasis. Bile acids, produced, like steroid hormones, from cholesterol, are inhibitors of 11β-HSD1 and some secondary bile acids (generated via gut bacterial activity) are directly metabolised by 11β-HSD1 (Perschel *et al*. 1991, Buhler *et al*. 1994, Odermatt *et al*. 2011, Penno *et al*. 2013, 2014). Importantly, the ability of bile acids to alter the gut microbiome has been demonstrated (Islam *et al*. 2011, Yokota *et al*. 2012), with potential clinical implications (Buffie *et al*. 2015). Thus, 11β-HSD1 has the potential to affect the microbiome via bile acid-mediated host–microbiome cross-talk as well as through glucocorticoid-regulated immune pathways.

In this study, we test the hypothesis that 11β-HSD1 influences the composition of the gut microbiome in a diet-dependent manner. To this end, we used high-throughput sequencing of amplified 16S rRNA gene sequences to quantify the composition of the gut microbiota from *Hsd11b1^Del1/De1l^* mice, with global knock-out of 11β-HSD1, and control C57Bl/6 mice. To explore possible interactions among 11β-HSD1 activity, the gut microbiome and diet, comparison between genotypes was made using mice fed on standard chow diet or on a high-fat/cholesterol-enriched ‘Western’ diet.

## Materials and methods

### Animals

Animal studies were carried out in strict accordance with the UK Home Office Animals (Scientific Procedures) Act, 1986 following approval by the University of Edinburgh Animal Welfare and Ethical Review Body. *Hsd11b1*^f/f^ mice (with a ‘floxed’ allele of the *Hsd11b1* gene) were generated by Artemis Pharmaceuticals (Cologne, Germany) onto a C57Bl/6 background (White *et al*. 2015). *LoxP* sites were placed flanking exon 3 of the mouse *Hsd11b1* gene, excision of which results in a null allele (here termed *Del1*) by out of frame splicing from exon 2 to exon 4. *Hsd11b1^Del1/De1l^* mice were generated by crossing *Hsd11b1*^f/f^ mice with *Hprt-Cre* transgenic mice (Tang *et al*. 2002), resulting in germline disruption of the *Hsd11b1* gene. *Cre^+^* offspring were bred with C57Bl/6 and *Cre^−^* offspring in which germline deletion of exon 3 of the *Hsd11b1* gene had occurred were backcrossed to C57Bl/6 for >5 generations. Animals were fed with standard chow (unless stated otherwise) and water *ad libitum.* Lights were on from 07:00 to 19:00, and temperature was maintained at 22°C. To avoid inter-animal variability that might be introduced due to differences in the stage of estrous in females, male mice were used in experiments.

Male *Hsd11b1^Del1/De1l^* mice and age-matched C57Bl/6 controls (10 weeks old) were singly housed in metabolic cages. Mice were either fed on standard chow (CRM diet: SDS, UK) or Western diet (WD, 41% fat with ~0.2% cholesterol; D12079B; Research Diets Inc., New Brunswick, NJ, USA) *ad libitum*, for 2 weeks (*n* = 6/group). Neither diet was associated with significant weight gain, measured over the second week (2-way ANOVA of body weight by genotype/time, chow diet: *P* = 0.06, Western diet: *P* = 0.8. Body weight gain on chow diet, C57Bl/6: 1.52 ± 0.23 g and *Hsd11b1^Del1/Del1^*, 1.19 ± 0.42 g. Body weight gain on Western diet: C57Bl/6: −0.33 ± 0.21 g and *Hsd11b1^Del1/Del1^*, −0.18 ± 0.25 g). All mice were killed by CO_2_ asphyxiation after a 4-h fast (08:00–12:00), and the contents of the caecum and colon were collected and frozen for subsequent DNA extraction.

### DNA extraction and sequencing

Genomic DNA was extracted from colon and caecal contents using a QIAamp DNA stool mini kit (with proteinase K used for lysis), as directed by the manufacturer (Qiagen). Extracted DNA was adjusted to 10 ng/μL and PCR amplified in duplicate (to control for PCR bias and error), following the protocol of Caporaso and coworkers (Caporaso *et al*. 2012) to target the variable V4 region of the 16S rRNA gene. PCR reactions (25 μL) contained 10 μL input DNA, 12.5 μL KAPA HiFi HS Ready Mix (Kapa Biosystems, Ltd, London, UK), 16S amplicon forward primer (5 pmol, Integrated DNA Technologies, Leuven, Belgium), 16S amplicon Golay barcoded reverse primer (5 pmol, Integrated DNA Technologies) and 1.0 μL H_2_O. PCR comprised an initial incubation at 98°C for 3 min followed by 20 cycles of 95°C for 20 s, 60°C for 30 s, 72°C for 45 s and a final extension of 72°C for 10 min. After amplification, the PCR products were diluted to 50 μL with 10 mM Tris–HCl pH 8.5 and cleaned using Ampure XP beads (Beckman Coulter Life Sciences, High Wycombe, UK) using a 1:1 bead to DNA ratio to remove excess primers, primer dimers and PCR reagents. Quality of amplicon libraries was assessed on a Bioanalyser High Sensitivity chip (Agilent Technologies) and quantification was done by qPCR using Kapa Library Quantification Kit for Illumina platforms (Kapa Biosystems, Ltd). Libraries were normalised and pooled in equimolar proportions prior to sequencing duplicate samples on an Illumina MiSeq platform using 150 base paired-end reads (v2 chemistry).

### Sequence assembly, filtering and taxonomic assignment

Paired-end reads were assembled into single contiguous sequences and filtered using Mothur (Schloss *et al*. 2009) (v1.33.3) to remove sequences that contained ambiguous base calls and those that were longer/shorter than 90% of the sequences in a sample. As an additional precaution, sequences with high similarity to the PhiX genome were also removed using blastn (Altschul *et al*. 1990) (*E*-value <1 × 10^−5^). Preliminary analysis indicated minimal technical variation between sequencing runs, and duplicate samples were therefore merged prior to further analysis. After merging duplicates and filtering, samples contained at least 489,266 assembled sequences (average 1,209,197 sequences).

Data were subsequently pooled across all samples, singletons (i.e. sequences appearing only once in the pooled dataset) were removed and the remaining data de-replicated to generate a set of unique sequences. Sequences representative of operational taxonomic units (OTUs) were then identified using the UPARSE pipeline (Edgar 2013) (usearch v8.0.1623), in which clustering was performed using the UPARSE-OTU algorithm and chimeras were identified using UCHIME in conjunction with the ChimeraSlayer reference database. All sequences were then assigned to an OTU using the USEARCH global algorithm (Edgar 2010) at a 97% similarity threshold. On average, 83.5% of sequences in a sample were successfully assigned to an OTU. Sequences that could not be assigned to an OTU were assumed to represent sequencing/assembly artefacts and were discarded from the study.

Taxonomic assignment of OTUs, OTU alignment and tree-building were performed using QIIME v1.9.0 (Caporaso *et al*. 2010*b*). The taxonomy of each OTU was determined using the RDP classifier v2.2 (Wang *et al*. 2007) in conjunction with the GreenGenes database, v13.5 (DeSantis *et al*. 2006) at a confidence threshold of 0.8. Representative OTU sequences were aligned using PyNAST (Caporaso *et al*. 2010*a*) and a neighbour-joining tree produced using FastTree (Price *et al*. 2009). Due to limitations in the ability of the V4 region to accurately identify closely related bacterial taxa, taxonomic classification of OTUs was restricted to the family level.

### Statistical analysis

For all groups, *n* = 6, with the exception of C57Bl/6 mice fed Western diet, where *n* = 5. Alpha diversity metrics Chao1 (Chao 1984) and phylogenetic distance (Faith & Baker 2006) were calculated using QIIME and rarefaction curves indicated that the minimum sequencing depth (489,266 assembled sequences) was sufficient to accurately capture diversity (Supplementary Fig. 1, see section on supplementary data given at the end of this article). For all other analyses, following McMurdie and Holmes (2014), counts of sequences assigned to each OTU were normalised using the variance stabilising (vst) transformation in the R package DESeq2 (Love *et al*. 2014). Statistical comparison of microbial diversity between samples was calculated on Euclidean distance matrices of vst-transformed count data using the permutational multivariate analysis of variance (PERMANOVA) approach of Anderson (2001), implemented in the R package vegan (Oksanen *et al*. 2013).

To identify individual OTUs with clearly defined differences between diets, genotypes or a strong diet–genotype interaction, Euclidean distance-based PERMANOVA tests were repeated using each individual OTU as a univariate response variable. For OTUs showing significance for a particular model term (*P* < 0.01), effect sizes (*η*^2^) and partial effect sizes (*η*_p_^2^) were calculated from sums of squares (SS) to reflect the variance attributable to the relevant term as a proportion of variance within the model, where:

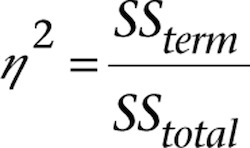


and:

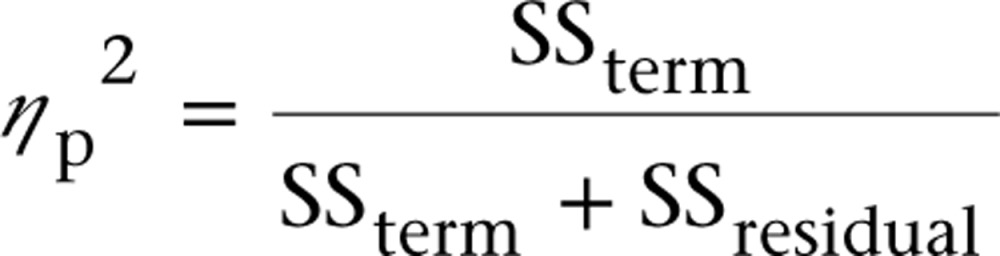


## Results

DNA was extracted from caecal, and colon contents were collected from *Hsd11b1^Del1/De1l^* and C57Bl/6 control mice fed either chow or Western diet. High-throughput sequencing of the V4 region of the bacterial 16S rRNA gene resulted in an average of 1.2 × 10^6^ amplicons per sample. Clustering amplicons at a 97% sequence similarity threshold identified 1152 operational taxonomic units (OTUs), of which 231 were present in all samples (Supplementary Fig. 2A). OTUs were assigned a taxonomic identity using public databases, and the relative abundance of reads assigned to each OTU was subsequently used as a basis on which to compare microbiome composition across samples.

### Changes in the gut microbiome as a consequence of diet and 11β-HSD1 activity

**Figure 1:**
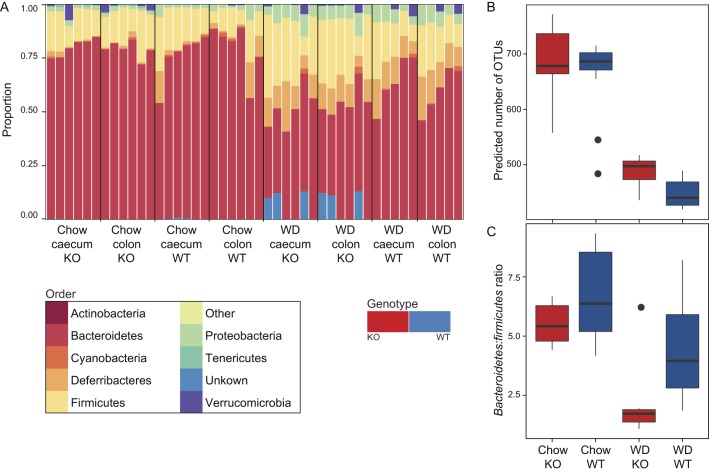
Gut microbiota of wild-type and *Hsd11b1^Del1/De1l^* mice fed Western and chow diets. (A) The relative proportion of bacterial sequence belonging to each of the dominant phyla detected in this study in *Hsd11b1^Del1/Del1^* (KO) and C57Bl/6 control (WT) mice. As no difference was observed between caecum and colon, these samples are merged in subsequent figures. (B) Chao1 estimates of the number of unique operational taxonomic units (OTUs) in the gut microbiome of *Hsd11b1^Del1/Del1^* (KO, red bars) and C57Bl/6 control (WT, blue bars) mice under different experimental conditions. Chao1 estimates were calculated after randomly down-sampling data so that all samples contain the same total number of OTU counts. (C) Ratio of the phyla Bacteroidetes and Firmicutes within each sample under different experimental conditions. Ratios were calculated on untransformed data.

First, we considered the effect of diet and genotype on the composition, richness and diversity of the gut microbiome. In mice fed a chow diet, the microbiome was dominated by bacteria belonging to the phyla *Bacteroidetes* and *Firmicutes* (Fig. 1A). Richness estimates for the chow diet indicated the presence of around 500–800 OTUs (median 690 OTUs, Fig. 1B). Consistent with previous studies (Kim *et al*. 2012), a Western diet increased the relative abundance of bacteria belonging to the phyla *Proteobacteria*, *Deferribateres* and *Firmicutes* (Hildebrandt *et al*. 2009) and significantly decreased both richness (median 475 OTUs, ANOVA, *F* = 185.56, *P* < 0.01, Fig. 1B) and the *Bacteroidetes*:*Firmicutes* ratio (ANOVA, *F* = 11.90, *P* = 0.03, Fig. 1C).

As observed previously, diet exerted a greater effect on composition of the gut microbiome than genotype (Carmody *et al*. 2015). Nonetheless, 11β-HSD1 deficiency resulted in an increase in the predicted number of OTUs (ANOVA, *F* = 5.16, *P* = 0.03, Fig. 1B) and a decrease in the ratio of *Bacteroidetes*:*Firmicutes* (ANOVA, *F* = 4.39, *P* = 0.05, Fig. 1C). Further changes in taxonomic abundance were observed at the family level (Supplementary Fig. 2). On a Western diet, 11β-HSD1 deficiency was associated with a number of OTUs that could not be confidently assigned to any phylum. These OTUs were not present in all samples, highlighting the potential for inter-individual variability in microbiome composition.

Statistical comparison of OTU diversity across samples was carried out using permutational multivariate analysis of variance (PERMANOVA). No significant effect of sample origin (caecum vs colon) was observed (*P*perm = 0.51). Accordingly, to avoid risk of type I error arising from repeated sampling from the same individual, data from caecal and colon samples for each animal were pooled to test for the effects of diet and genotype on diversity.

**Figure 2:**
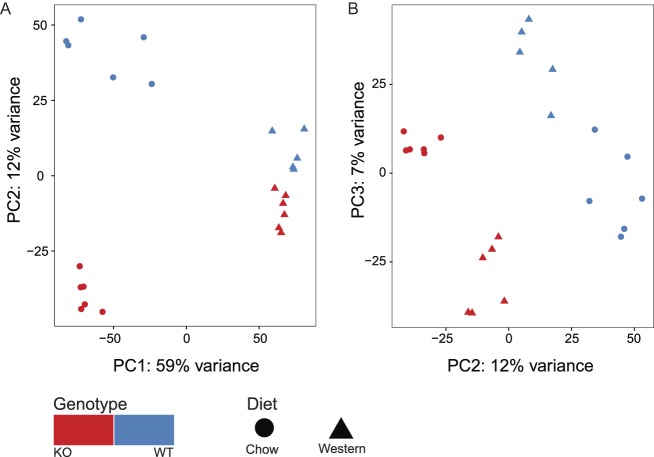
Principal components analysis (PCA) ordination showing the effect of diet and 11β-HSD1 activity on the composition of the gut microbiota. Plots show (A) the first, second and (B) second and third axes of a PCA based on the abundance of the 1152 OTUs detected in this study. Red symbols: *Hsd11b1^Del1/Del1^* (KO) mice, blue symbols: C57Bl/6 control (WT) mice. For all groups, *n* = 6, with the exception of C57Bl/6 mice fed Western diet, where *n* = 5.

Statistical analysis showed that both diet and genotype significantly altered the diversity of the gut microbiome (*P*perm < 0.01, Table 1). Moreover, PERMANOVA tests revealed a significant interaction term, indicating that the effect of genotype was dependent upon the diet (*P*perm < 0.01, Table 1). Principal components analysis (PCA) revealed that diet exerted the strongest influence on microbial diversity, accounting for 59% of the observed variance in OTU abundance (PC1, Fig. 2A). Subsequent principal component axes indicated that genotype exerted a strong influence on microbial diversity, with 19% of the variation in diversity explained by differences between *Hsd11b1^Del1/De1l^* and control microbiomes in mice fed a chow or Western diet (PC2 and PC3, respectively, Fig. 2B).

**Table 1:** Results of permutational multivariate analysis of variance (PERMANOVA) test comparing composition of the gut microbiota across diets (chow vs Western) and genotypes (C57Bl/6 vs *Hsd11b1^Del1/Del1^*). The test was based on vst-transformed counts of OTU abundance. For all groups, *n* = 6, with the exception of C57Bl/6 mice fed Western diet, where *n* = 5.

	***Df***	**Sums of Sqs**	**Mean Sqs**	***F***	***P***
Diet	1	107,517	107,517	35.64	0.00001
Genotype	1	22,727	22,727	7.534	0.0014
Diet × genotype	1	17,496	17,496	5.80	0.0027
Residual	19	57,318	3017	0.28	

### Identifying bacterial taxa whose abundance is dependent on diet and 11β-HSD1 activity

**Figure 3:**
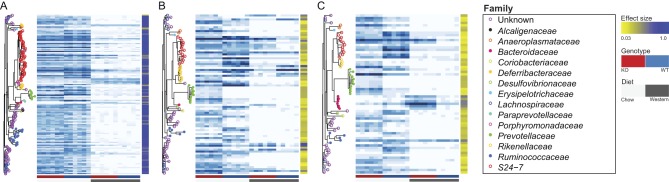
Heat maps depicting the relative abundance of OTUs for which (A) variation in abundance is dependent on diet, (B) variation in abundance is dependent on genotype, (C) variation in abundance as a consequence of genotype is dependent on diet (i.e. a diet × genotype interaction). For each contrast, data are only shown for the top 25% of significant OTUs ranked by the partial effect size (*η*_p_^2^) of the relevant model term in univariate analysis of variance tests for differences in abundance between experimental conditions. Phylograms to the left of each heat map depict the phylogenetic relatedness between OTUs. Colour scales to the right of each OTU depict the proportion of the total variance (*η*^2^) that could be explained by the relevant model term. Colour bars beneath the heat maps indicate genotype (*Hsd11b1^Del1/Del1^* (KO) mice: red, C57Bl/6 control (WT) mice: blue) and diet (chow: light grey, Western: dark grey). For all groups, *n* = 6, with the exception of C57Bl/6 mice fed Western diet, where *n* = 5.

Next, we sought to identify the main bacterial taxa responsible for driving differences in diversity that resulted from changing diet or 11β-HSD1 activity. Effect sizes were calculated (see ‘Methods’ section) and for each OTU, they were used to represent the proportion of observed variation in abundance that could be explained by diet, genotype or a diet × genotype interaction (Supplementary Fig. 3). The 25% of OTUs with the largest effect size for a particular model term are depicted in Fig. 3.

Consistent with multivariate analysis, effect sizes (*η*^2^) demonstrated that diet frequently accounted for a greater proportion of variation in individual OTU abundance than either genotype or a diet × genotype interaction (Fig. 3A, B and C). Of those OTUs that significantly (*P*perm < 0.01) altered abundance in response to diet, the majority (494 out of 651) showed a decrease in relative abundance on the Western diet (Fig. 3A). This was evident for a number of OTUs belonging to the families *S4-7*, *Prevotellaceae*, *Ruminococcaceae* and *Lachnospiraceae*. However, the opposite was observed for OTUs belonging to the families *Deferribacteraceae* and *Porphyromonadaceae*, as well as a small number of OTUs belonging to the family *S24-7*.

Genotype had a significant effect on 327 OTUs (*P*perm < 0.01), and this effect was less one-sided (Fig. 3B), with some families, such as the *Anaeroplasmataceae* and *Prevotellaceae*, showing greater relative abundance in *Hsd11b1^Del1/De1l^* microbiomes, and others, such as the *Rikenellaceae*, showing greater relative abundance in wild-type microbiomes. Notably, many families affected by genotype showed changes in abundance that were moderated by diet, indicating that the effect of 11β-HSD1 activity on individual taxa was diet dependent. When directly considering OTUs for which an interaction between diet and genotype altered relative abundance (Fig. 3C), effect sizes indicated that this interaction had strongest explanatory power for bacteria belonging to the family *Bacteroidaceae*.

**Figure 4:**
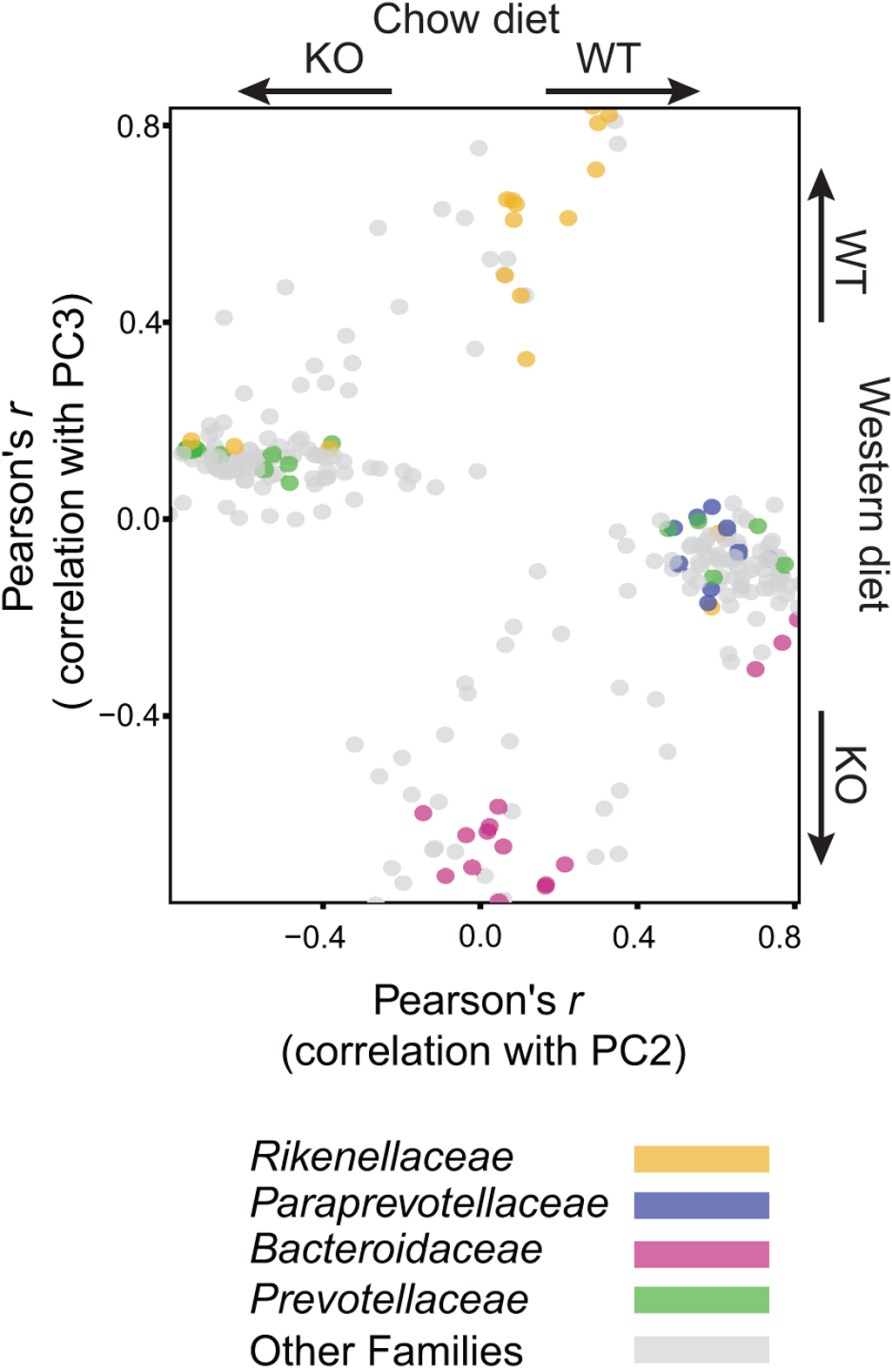
Bacterial taxa responsible for differences in diversity between the gut microbiota of C57Bl/6 (WT) and *Hsd11b1^Del1/De1l^* (KO) mice on different diet backgrounds. Each data point represents a single OTU and data are only shown for OTUs that have a significant diet × genotype interaction (*P* < 0.01) in univariate analysis of variance tests for differences in abundance between experimental conditions. The *x* axis shows the Pearson correlation between the abundance of an OTU and the position of samples along the principal component axis in Fig. 2 that separates C57Bl/6 and *Hsd11b1^Del1/De1l^* mice on a chow diet (PC2). The *y* axis shows the Pearson correlation between the abundance of an OTU and the position of samples along the principal component axis in Fig. 2 that separates C57Bl/6 and *Hsd11b1^Del1/De1l^* mice on a Western diet (PC3). A strong positive correlation indicates that an OTU contributes to the diversity that distinguishes WT mice, a strong negative correlation indicates that an OTU contributes to the diversity that distinguishes KO mice. The colour of each data point depicts families that show strong trends that are restricted to different diet backgrounds. Changes in the relative abundance of these families in response to diet and 11β-HSD1 deficiency can be seen in Supplementary Fig. 2B. For all groups, *n* = 6, with the exception of C57Bl/6 mice fed Western diet, where *n* = 5.

To investigate further how diet affected the microbiome response to 11β-HSD1 deficiency, we used Pearson’s method to correlate changes in the relative abundance of each OTU showing a significant diet × genotype interaction with the position of samples along the PC axes separating control and *Hsd11b1^Del1/De1l^* microbiomes on different diet backgrounds (as depicted in PC2 and PC3 in Fig. 2B). As expected, the majority of OTUs gave a strong Pearson’s *r* score, demonstrating strong correlation with one or the other of the explanatory PC axes (Fig. 4). Notably, however, overlaying information about taxonomic classification showed that many OTUs belonging to the families *Prevotellaceae* and *Paraprevotellaceae* displayed greater relative abundance in *Hsd11b1^Del1/De1l^* microbiomes and C57Bl/6 microbiomes, respectively, on a chow diet background. By contrast, OTUs belonging to the families *Bacteroidaceae* and *Rikenellaceae* displayed greater relative abundance in *Hsd11b1^Del1/De1l^* microbiomes and C57Bl/6 microbiomes, respectively, on a Western diet background. Although most OTUs showed changes in relative abundance in response to altered diet/genotype that were consistent with other members of their family, several showed alternative patterns of abundance. For example, three OTUs belonging to the family *Bacteroidaceae* showed greater relative abundance in wild-type mice on a chow diet background, whereas the majority of OTUs belonging to this family showed altered response to genotype only on a Western diet background. Such patterns of abundance potentially reflect functional divergence of taxa below the family level, not detected in this study because of the limited taxonomic resolution provided by sequencing the V4 region. Taken together, however, these results demonstrate that 11β-HSD1 activity exerts an effect on the abundance of discrete bacterial taxa that is diet dependent and broadly consistent at family level.

## Discussion

Here, we demonstrate that the enzyme 11β-HSD1 acts to alter the composition of the mouse gut microbiome, and does so in a diet-dependent manner. Most notably, we show that 11β-HSD1 deficiency increases the relative abundance of the families *Prevotellaceae* on a chow diet and *Bacteroidaceae* on a Western diet. These families contain genera associated with inflammatory and cardiovascular disease, respectively. They also contain the principal genera that define common bacterial profiles (enterotypes) found within the human gut (Arumugam *et al*. 2011), which may themselves be diet related (Wu *et al*. 2011).

The family *Prevotellaceae* is associated with inflammatory disease in both humans and mouse models. In a study of patients with new-onset untreated rheumatoid arthritis, the presence of *Prevotella copri* correlated strongly with disease occurrence (Scher *et al*. 2013). We have previously reported that chow-fed 11β-HSD1-deficient mice show increased severity of acute inflammation, including in an experimental model of arthritis (Coutinho *et al*. 2012). Our findings here raise the possibility that this is related to the increased relative abundance of bacteria belonging to the *Prevotellaceae* in their gut microbiota. *P. copri* is also associated with more severe inflammation in animal models of colitis. Mice colonised with *P. copri* showed more severe colitis when exposed to dextran sulfate sodium (DSS) (Scher *et al*. 2013), probably mediated via induction of the cytokine CCL5, which exaggerates DSS-induced colitis (Elinav *et al*. 2011). 11β-HSD1 gene expression is increased in rodents during DSS-induced colitis and also in patients with inflammatory bowel disease (Zbankova *et al*. 2007, Stegk *et al*. 2009). However, this is interpreted as an anti-inflammatory mechanism to increase local glucocorticoid exposure, and whether reduced expression or inhibition of 11β-HSD1 can predispose to colitis has not been tested.

A high-fat/cholesterol diet profoundly alters the gut microbiota (Hildebrandt *et al*. 2009, David *et al*. 2014). High-fat/cholesterol diets also induce intestinal inflammation, which is dependent upon the gut microbiota (Ding *et al*. 2010, Kim *et al*. 2012, Progatzky *et al*. 2014) and, at least for high-fat diet, has been associated with a decrease in the *Bacteroidetes*-to-*Firmicutes* ratio (Kim *et al*. 2012). Here, we show a dramatic alteration in the gut microbiota in mice fed a high-fat Western diet, including an increase in the *Bacteroidetes*-to-*Firmicutes* ratio that is consistent with the previous data. In addition, we show a smaller, but nonetheless significant, effect of genotype. Interestingly, and in contrast to chow-fed mice, Western diet-fed 11β-HSD1-deficient mice show a higher relative abundance of the family *Bacteroidaceae*. Whether this is beneficial or not remains to be determined. However, we have previously reported that 11β-HSD1 deficient mice fed a high-fat diet show reduced inflammatory signalling in mesenteric adipose tissue, a depot that contains the lymph nodes that drain the colon (Wamil *et al*. 2011), suggesting a lower level of intestinal inflammation. In the future, it will be important to determine if the altered gut microbiota observed in Western diet-fed 11β-HSD1-deficient mice elicits milder intestinal inflammation.

Recent, large-scale studies of human cohorts have indicated that the presence and increased relative abundance of *Bacteroidaceae* (including the genus *Bacteroides*) is associated with a significant decrease in plasma triglyceride levels (Fu *et al*. 2015). Relative abundance of *Bacteroides* also correlates negatively with body mass index (Goodrich *et al*. 2014, Fu *et al*. 2015). Intriguingly, 11β-HSD1 deficiency reduces plasma triglyceride levels and attenuates weight gain in mice fed a high-fat diet (Morton *et al*. 2004). We have also reported that 11β-HSD1 deficiency reduces plasma triglyceride levels and is protective against atherosclerosis in Western diet-fed atherosclerosis-prone *Apoe*^−/−^ mice (Kipari *et al*. 2013). However, in a similar study using a different line of 11β-HSD1 knockout mice, the reduction in plasma triglyceride levels was only observed in female mice (García *et al*. 2013). Nevertheless, the coincidence of 11β-HSD1 deficiency, *Bacteroidaceae* abundance and reduced plasma triglyceride levels highlights a potential causative relationship that is deserving of further study.

The mechanisms that underlie the diet-specific effects of 11β-HSD1 deficiency upon the gut microbiome are currently unclear. However, two possibilities are likely: alterations in glucocorticoid signalling or alterations in bile acid composition and/or signalling. 11β-HSD1 is an important modulator of intracellular glucocorticoid levels, with glucocorticoids being one of the most potent regulators of immune cell function and phenotype. 11β-HSD1 is expressed and functional in a variety of immune cells including macrophages (Gilmour *et al*. 2006, Zhang & Daynes 2007), dendritic cells (Freeman *et al*. 2005, Soulier *et al*. 2013), mast cells (Coutinho *et al*. 2013) and lymphocytes (Zhang *et al*. 2005), any of which could contribute to tolerance of gut microbes (Mortha *et al*. 2014). Altered intracellular glucocorticoid levels as a result of 11β-HSD1 may therefore alter the local immune environment within the gut, with differential consequences for the microbiome, dependent upon diet. Tissue-specific disruption of *Hsd11b1* in these cell types could address the possible role of 11β-HSD1 in tolerance to gut microbes.

11β-HSD1 may also mediate host–microbiome interactions in a diet-specific manner via its role in bile acid homeostasis. High-fat diets both increase the quantity and alter the composition of bile acids in the gut (Ridlon *et al*. 2014), and bile acids have antimicrobial properties that make them important regulators of the gut microbiome (Begley *et al*. 2005). High-fat diets can therefore promote expansion of bile-tolerant bacteria, for example *Bacteroides*, which may in turn alter the synthesis of secondary bile acids. Microbial metabolism of bile acids in the intestines is a major determinant of bile acid pool size and composition (Begley *et al*. 2005, Sayin *et al*. 2013). Previously, we have reported preliminary data showing that 11β-HSD1 deficiency in mice impairs post-prandial bile acid release and alters the profile of bile acids in bile fluid, with a switch from the predominance of 7β-hydroxylated to 7α-hydroxylated bile acids (Opiyo *et al*. 2014). Whether 11β-HSD1 itself directly converts 7α-hydroxylated to 7β-hydroxylated bile acids is currently unknown, but several oxysterols with a keto moiety at the 7 position on the B ring are substrates for 11β-HSD1 (Odermatt & Nashev 2010). Importantly, this includes the secondary bile acid 7-oxolithocholic acid (7-oxoLCA), converted by 11β-HSD1 into chenodeoxycholic acid (CDCA) (Odermatt *et al*. 2011), a potent activator of the nuclear bile acid receptor, also known as the farnesoid X receptor (FXR). It is therefore plausible that 11β-HSD1 influences the composition of the gut microbiome by altering bile acid profile via its role in the synthesis of 7α-hydroxylated bile acids. This merits future investigation.

In conclusion, we show that the effect of 11β-HSD1 activity on the gut microbiome is diet dependent and alters the relative abundance of bacteria relevant to human clinical studies. Two plausible mechanisms for 11β-HSD1 mediation of host–microbiome interaction involve immune system regulation and bile acid homeostasis, and further functional studies are required to reveal the precise role of the gut microbiome in shaping the 11β-HSD1 deficiency phenotype. With the development of selective inhibitors of 11β-HSD1 for the treatment of metabolic disease and age-related cognitive decline (Anderson & Walker 2013, Chapman *et al*. 2013), our studies could provide new insights into how diet may influence the outcome of therapeutic 11β-HSD1 inhibition.

## Supplementary data

This is linked to the online version of the paper at http://dx.doi.org/10.1530/JOE-16-0578.

## Declaration of interest

J R S holds patents on selective 11β-HSD1 inhibitors.

## Funding

This work was supported by a Wellcome Trust Programme grant (083184; J R S, K E C). M N O was supported by a scholarship from the College of Medicine and Veterinary Medicine, University of Edinburgh. Edinburgh Genomics (University of Edinburgh) is partly supported through core grants from NERC (R8/H10/56), MRC (MR/K001744/1) and BBSRC (BB/J004243/1). The Computational Genomics Analysis and Training Centre (CGAT) was supported by the Medical Research Council (G1000902).

## Author contribution statement

K E C and A H contributed to study conception; J S J, A H, K E C, K G and J R S designed the study. J S J, M N O and M T executed the study. J S J, A H and K E C contributed to interpretation and J S J, K E C and A H prepared the manuscript.
